# Dissociating sensitivity from bias in the Mini Profile of Music Perception Skills

**DOI:** 10.1121/10.0021096

**Published:** 2023-09-25

**Authors:** Kelly L. Whiteford, Pui Yii Goh, Kara L. Stevens, Andrew J. Oxenham

**Affiliations:** Department of Psychology, University of Minnesota, Minneapolis, Minnesota 55455, USA whit1945@umn.edu, goh00002@umn.edu, stev0566@umn.edu, oxenham@umn.edu

## Abstract

The Mini Profile of Music Perception Skills (Mini-PROMS) is a rapid performance-based measure of musical perceptual competence. The present study was designed to determine the optimal way to evaluate and score the Mini-PROMS results. Two traditional methods for scoring the Mini-PROMS, the weighted composite score and the parametric sensitivity index (*d′*), were compared with nonparametric alternatives, also derived from signal detection theory. Performance estimates using the traditional methods were found to depend on response bias (e.g., confidence), making them suboptimal. The simple nonparametric alternatives provided unbiased and reliable performance estimates from the Mini-PROMS and are therefore recommended instead.

## 1. Introduction

The question of whether musical training is associated with benefits outside the domain of music is of interest to both scientists and the broader community (Jakobson and Cuddy, 2019). The results from such studies have often remained difficult to interpret for two main reasons. First, most studies have been cross-sectional, comparing expert musicians with non-musicians, meaning that no causal effect of musical training can be inferred (McKay, 2021; Schellenberg, 2020). Second, musical ability is often assessed via self-reported measures, such as years of musical training, age of onset of musical training, total number of hours of practice, and/or self-assessed musical status or sophistication (Müllensiefen *et al.*, 2014; Zhang *et al.*, 2020). Such measures are convenient and quick, but they may not capture important aspects of musical expertise, such as the quality of the musical training, the amount of informal musical experience or exposure, or the degree of innate musical ability (Correia *et al.*, 2023; Mankel and Bidelman, 2018). More objective, performance-based measures of musicianship can help to overcome this second limitation.

Performance-based measures of musicality date back over a century (Seashore, 1919), with contributions continuing up to the present [for a review, see Zentner and Gingras (2019)]. Of these, the Profile of Music Perception Skills (PROMS) [∼1 h duration (Law and Zentner, 2012)] and its shorter variants, Brief PROMS [∼25 min (Law and Zentner, 2012)], PROMS-Short [∼30 min (Zentner and Strauss, 2017)], Mini-PROMS [∼15 min (Zentner and Strauss, 2017)], and Micro-PROMS [∼10 min (Strauss *et al.*, 2023)], have proved popular. In all PROMS versions, participants are presented with one melody, twice in a row, followed by a third melody, and then asked to judge whether the third melody is the same or different from the first two melodies, using one of five options: “definitely different,” “probably different,” “I don't know,” “probably same,” and “definitely same.” All versions of the PROMS have demonstrated evidence of validity and reliability and can be administered online (Musemap, 2023), making them accessible to a wide population of participants.

While the PROMS has a number of strengths for use in scientific research [e.g., Kunert *et al.* (2016)], the interpretation of the test results rests on the assumption that the method of scoring the PROMS reflects true sensitivity to changes in melodic sequences. However, the current recommended method for scoring the PROMS is to allot full credit to correct and confident responses, but only half credit to correct but less confident responses (Law and Zentner, 2012), meaning that the score conflates sensitivity with confidence, thereby potentially introducing the influence of bias. Simply calculating the percentage of correct responses and ignoring the confidence ratings can help, but does not solve the additional problem that percent correct varies with response bias and tends to underestimate performance when bias is present [e.g., Henry and McAuley (2013)].

An alternative, more precise method of quantifying sensitivity is to use methods from signal detection theory (SDT) to calculate the sensitivity index, *d′* (Macmillan and Creelman, 2005). Although this measure has been used for scoring the PROMS (Law and Zentner, 2012; Zentner and Strauss, 2017), its advantage as a bias-free measure is contingent on the underlying internal representations of the same and different trials having equal variances (Macmillan and Creelman, 2005; Stanislaw and Todorov, 1999), an assumption that has not been tested for the PROMS.

The purpose of the present study was to compare current scoring methods for PROMS with alternatives that do not rely on parametric assumptions. We used the Mini-PROMS due to its short test duration (15 min), the high relevance of the subtests measured for musicianship researchers (melody, tuning, accent, and tempo), and its equal proportion of same and different trials (unlike the Micro-PROMS, which uses 75% different trials, making it more susceptible to effects of response bias). We used the confidence ratings to calculate empirical receiver operating characteristic (ROC) curves and tested the equal variance assumption.

## 2. Methods

### 2.1 Participants

All participants were recruited from the Research Experience Program at the University of Minnesota, provided informed consent, and received course credit for their time. A total of 75 participants (52 female, 23 male) between the ages of 18 and 29 years (mean = 20.4, SD = 2.3) completed the full online experiment. An additional 38 participants were excluded for failing the attention check (n = 22), self-reporting audio issues during the experiment (n = 20), and/or because their total experiment duration exceeded 30 min (n = 11). All participants reported having normal hearing. The procedures were approved by the Institutional Review Board of the University of Minnesota.

### 2.2 Stimuli and procedures

Tasks were administered remotely using the PROMS online interface (Law and Zentner, 2012; Zentner and Strauss, 2017) in the order described below.

#### 2.2.1 Survey questions and attention check

Participants answered survey questions related to demographic information, hearing status, and musical background, including their total years of musical training, defined as “group and private lessons or practicing on your own.” They then completed the attention check, which ensured that participants had functioning audio capabilities on their remote setup, that the stimuli were audible, and that the participants were attending to the stimuli. Participants were first presented with a short melody and asked to adjust their volume to a comfortable level. The participants were then presented with four trials, each consisting of a sequence of 1-kHz tones of 400-ms duration (including 50-ms raised-cosine onset and offset ramps), separated by 500-ms of silence. Participants were instructed to report the number of tones within each trial by selecting their answer from a dropdown menu, with options ranging from zero to nine tones. To pass the attention check, participants had to answer at least three of four trials correctly. Because there were 10 possible answers for each trial, the probability of passing the attention check by chance was very low (p = 0.0037).

#### 2.2.2 Mini-PROMS

The Mini-PROMS was administered as described in Zentner and Strauss (2017) and consisted of four subtests: Melody, Tuning, Tempo, and Accent. In each trial, participants were presented with a single melody, repeated once, followed by a third melody. Participants were instructed to determine whether the third melody was the same or different compared to the previous two by selecting the following options from a dropdown menu: “definitely different,” “probably different,” “I don't know,” “probably same,” and “definitely same.” Trials that were different either varied in melodic line (Melody), were out of tune (Tuning), varied in beats per minute (Tempo), or had a level increase applied to one or more notes (Accent). For each subtest, half of the trials were different. The Melody and Accent subtests consisted of 10 trials each, while the Tuning and Tempo subtasks were 8 trials each.

### 2.3 Statistical analyses

All data were analyzed in matlab 2020b. All *p*-values are from two-tailed tests unless otherwise stated. The nonparametric bootstrap analysis was conducted by resampling the slopes of the z-transformed ROC (zROC) with replacement for 10 000 iterations using the *datasample* function. The 95% confidence intervals (CI) were constructed using the percentile method (Efron and Tibshirani, 1998).

## 3. Results

### 3.1 Composite scores versus d′

Performance was first scored using the traditional composite method, as described by Law and Zentner (2012) and Zentner and Strauss (2017), by assigning 1 point to correct and confident responses (i.e., “definitely same” responses for same trials and “definitely different” responses for different trials), 0.5 points to correct but less confident responses (“probably same” and “probably different”), and 0 points to incorrect or “I don't know” responses, and then summing the total. Using this method, it is only possible to receive the maximum 36 points (100%) if all trials are answered correctly and confidently, whereas someone who is correct across all trials but never answers confidently would only receive 18 points (50%).

As a comparison, SDT was used to calculate sensitivity (*d′*), where correctly identified “different” trials were considered hits, and false alarms were when the participant incorrectly responded “different” to same trials, irrespective of confidence rating,

$$d^{\prime }=z\left(H\right)-z(FA),$$ (1)

where *H* corresponds to the proportion of hits, *FA* corresponds to the proportion of false alarms, and *z* is the inverse of the cumulative normal probability distribution function. Note that this calculation differs from that used by Law and Zentner (2012), who weighted scores based on confidence before calculating *d′*, again conflating sensitivity with bias. Proportions of 0 and 1 were transformed to 1/2 *N* and 1–1/2 *N*, respectively, where *N* is the total number of trials on which the proportion is based [e.g., *N* = 5 for the melody subtest and *N* = 4 for the tempo subtest (Macmillan and Creelman, 2005)].

**Fig. 1.**
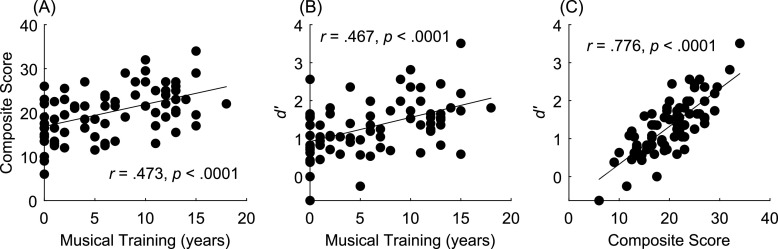
Years of musical training significantly correlated with (A) the Mini-PROMS composite score and (B) sensitivity, and (C) these two measures were strongly correlated with each other. However, the composite score will misrepresent true sensitivity in people with very poor confidence and high sensitivity or very high confidence but poor sensitivity, as denoted by the wide spread in *d′* values for participants with moderate composite scores.

Table 1 compares the average performance in the present study with that of Zentner and Strauss (2017). The composite scores in the present study are similar but slightly lower than in Zentner and Strauss (2017), whereas the *d′* values are higher. Both the composite scores and *d′* were moderately correlated with years of musical training in the present study [composite: *r* = 0.473, *p* < 0.0001; *d′*: *r* = 0.467, *p* < 0.0001; Fig. 1(A) and Fig. 1(B)], consistent with past studies (Law and Zentner, 2012; Zentner and Strauss, 2017), confirming adequate criterion validity. Although *d′* and composite scores were highly correlated with each other (*r* = 0.776, *p* < 0.0001), Fig. 1(C) illustrates a large spread of *d′* values across individuals with similar moderate composite scores. For example, one participant with a composite score of 20.5 out of 36 could be interpreted as having mediocre performance, but their actual sensitivity of *d′* = 2.44 placed them among the five most sensitive of the 75 participants. Another participant with an only slightly lower composite score of 17.5 was completely incapable of discriminating between same/different melodies (*d′* = 0), meaning they either have very poor melody perception or they were not complying with task instructions. Further examination of this participant's raw data indicated that they only responded, “definitely different” and “probably different” to one trial each. They had a single H and a single FA, resulting in a *d′* = 0 but a moderate composite score (as most of the “same” answer trials were correctly answered confidently).

**Table 1.** Average composite scores, *d′*, and *c*. Higher *d′* indicates better sensitivity, whereas higher *c* indicates a tendency for participants to respond “same” more often than “different.” Numbers in parentheses show standard deviations (SD).

	Composite score		*d′*		*c*
Subtest	Zentner and Strauss (2017)	Current work	Zentner and Strauss (2017)	Current work	Current work
Melody	6.43 (1.8)	5.49 (1.8)	0.640 (0.21)	1 (0.81)	−0.038 (0.46)
Tuning	5.44 (1.6)	4.43 (1.7)	0.91 (0.33)	1.07 (0.77)	0.519 (0.37)
Tempo	6.15 (1.4)	4.77 (1.7)	1.10 (0.26)	1.40 (0.80)	0.134 (0.37)
Accent	6.50 (1.7)	5.33 (1.8)	0.730 (0.24)	1.02 (0.75)	0.265 (0.49)
Composite Mini-PROMS	24.6 (4.8)	20 (5.6)	0.680 (0.10)	1.32 (0.72)	0.263 (0.41)

### 3.2 Response bias and musical training

The propensity to respond “same” more often than “different,” or vice versa, is known as response bias and can be quantified using the following formula (Macmillan and Creelman, 2005):

$$c=-\frac{1}{2}[z\left(H\right)+z\left(FA\right)].$$ (2)

Response bias was calculated for each subtest separately as well as the total Mini-PROMS. Average response biases are presented in Table 1. The total Mini-PROMS had *c* = 0.263, indicating that participants tended to respond “same” more often than “different.” Within the subtests, tuning and accent had the highest response bias, followed by tempo, with melody demonstrating very little response bias.

**Fig. 2.**
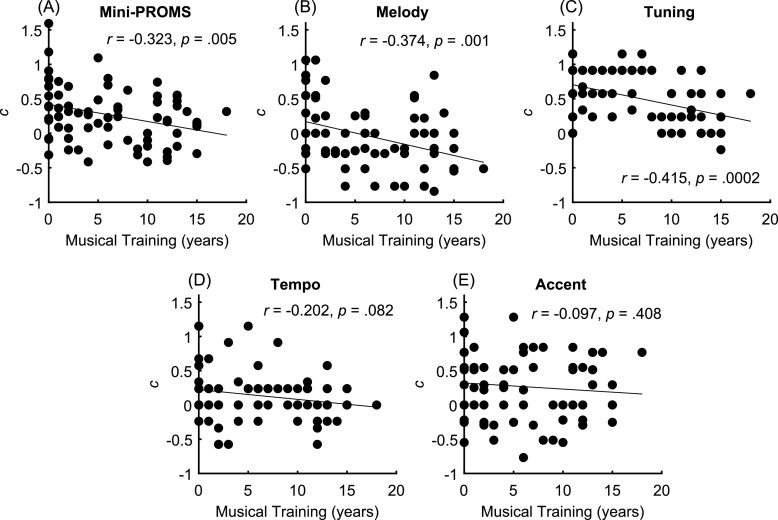
Response bias significantly decreased (i.e., decreased “same” responses) with years of musical training for (A) the total Mini-PROMS as well as the (B) melody and (C) tuning subtests but not the (D) tempo or (E) accent subtests.

Figure 2 illustrates wide variation in response bias across participants. Correlations between years of musical training and the Mini-PROMS were conducted using Bonferroni correction for five comparisons (*α* = 0.01). Response bias for the total Mini-PROMS was negatively correlated with years of musical training (r = −0.323, p = 0.005), meaning the tendency to respond “same” more often than “different” decreased with musical training. Similar significant correlations were found for the melody (r = −0.374, p = 0.001) and tuning (r = −0.415, p = 0.0002) subtests but not the tempo (r = −0.202, p = 0.082) or accent (r = −0.097, p = 0.408) subtests.

### 3.3 Empirical ROCs and the equality of variances assumption

**Fig. 3.**
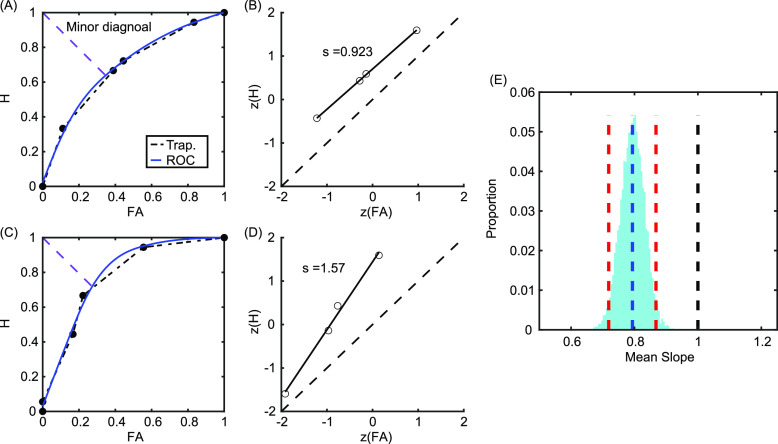
Empirical ROCs [(A) and (C)] and zROCs [(B) and (D)] for two example participants. When the signal and noise distributions have similar standard deviations (top row), the ROC (blue line) is relatively symmetric along the minor diagonal (purple dashed line), and the slope of the z-transformed ROC line (solid black line) approximates 1. When the signal and noise have different standard deviations (bottom row), the ROC is asymmetric and the slope of the zROC differs from 1. Left column: Black dashed line represents the ROC as calculated using the trapezoidal method. Middle column: Dashed line is the major diagonal. (E) Bootstrapped probability distribution for average zROC slopes. The blue-dashed lines correspond to the actual average slope, the black-dashed line corresponds to what the slope should be if equality of variances were satisfied, and the red-dashed lines are 95% CIs.

The *d′* measure calculated from proportions of H and FA is a bias-free estimate of sensitivity if the internal representations of the same and different trials have similar underlying distributions (i.e., equality of variances). This assumption was tested directly by plotting empirical ROC curves for each individual subject, with the cumulative FA on the *x* axis and cumulative H on the *y* axis for each confidence rating. Qualitatively, the ROC curve should have a symmetric shape around the minor diagonal if the distributions have similar variances. Figure 3 shows the ROC curves of two example participants, one where the equality of variances assumption is met [Figs. 3(A), 3(B)] and one where the assumption is clearly violated [Figs. 3(C), 3(D)]. Each data point along the ROC curve represents an estimate of sensitivity as a listener shifts their criterion. If the equality of variances assumption is satisfied, the *d′* at each criterion (i.e., confidence rating) should be similar (i.e., *d′* should be independent of response bias). In terms of the zROC, this implies that each datapoint should be an equal distance above the major diagonal. Qualitatively, this expectation is met for the participant where the assumption is satisfied [Fig. 3(B)] but not for the participant where the assumption is violated [Fig. 3(D)].

The degree to which the assumption of equality of variance is met or violated can be quantitatively estimated via the slope of the zROC function, which corresponds to the ratio of the standard deviations of the signal and noise distributions. If the equality of variances holds, the slope should be approximately equal to 1. This slope was calculated for each participant using linear regression (i.e., the line of best fit that minimizes the mean-squared error of the *y* axis). One participant was excluded from this analysis due to having only one data point on the ROC curve, as they responded confidently to every trial. The slopes ranged from 0 to 1.57, with an average slope of 0.794. Nonparametric bootstrapping was used to derive 95% confidence intervals by resampling the 74 participant slopes with replacement. The mean across each of the 10 000 resampled iterations was used to construct the bootstrapped distribution [Fig. 3(E)]. The CI [0.719, 0.863] does not encompass 1, indicating that the equality of variances assumption is violated. Therefore, a non-parametric estimate of sensitivity is required.

### 3.4 Non-parametric estimates of sensitivity

One non-parametric estimate of sensitivity is the area under the ROC curve (AUC). A common and relatively simple method for calculating the AUC is to use linear extrapolation (Macmillan and Creelman, 2005; Pollack and Hsieh, 1969; Stanislaw and Todorov, 1999), by connecting each point on the ROC with a straight line and then calculating the area underneath the straight lines (left column in Fig. 3; black dashed lines). Calculating the AUC using this method will tend to underestimate sensitivity from the true ROC (Fig. 3; blue line), but it is more straightforward than individually fitting a curvilinear function for each participant, as each individual may require a different type of function depending on the shape of their ROC.

A second method is to use a nonparametric estimation of *d′*. When parametric assumptions are met [Fig. 3(B)], *d′* is equivalent to the distance between the major diagonal and the zROC line, both horizontally and vertically (Macmillan and Creelman, 2005). When parametric assumptions are violated [Fig. 3(D)], the horizontal (*d′*_1_) and vertical (*d′*_2_) estimates of *d′* differ. A commonly used compromise between the two is to calculate *d_a_* using the following formula (Macmillan and Creelman, 2005; Simpson and Fitter, 1973):

$$d_{a}={\left(\frac{2}{1+s^{2}}\right)}^{1/2}d_{2}^{\prime },$$ (3)

where s is the slope of the zROC and *d′*_2_ is the zROC value when z(FA) = 0.

One drawback to *d_a_* when equality of variances is violated is that the slope of the zROC will differ depending on whether the line of best fit minimizes the mean-squared error of the cumulative proportion of hits (*y* axis) or the false alarms (*x* axis). A third measure, *d′_p_*, uses principal component analysis (PCA) to find the line of best fit that minimizes the mean-squared error of both the x- and *y*-axes (Vokey, 2016). The mathematical shortcut is simple to calculate,

$$d_{p}^{\prime }={\bar{z}}_{H}-{\bar{z}}_{FA},$$ (4)

where ${\bar{z}}_{H}$ and ${\bar{z}}_{FA}$ are the average of the z-transformed cumulative proportion of H and FA across all confidence ratings [e.g., for the four data points in Fig. 3(B), the mean of the *y* axis values ( ${\bar{z}}_{H}$) and the mean of the *x* axis values ( ${\bar{z}}_{FA}$)].

**Fig. 4.**
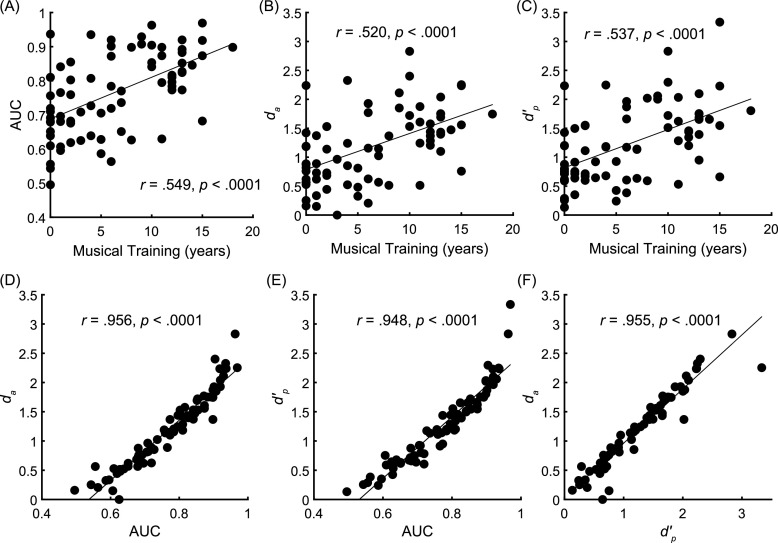
Correlations between nonparametric measures of Mini-PROMS performance demonstrate (A)–(C) adequate construct validity and (D)–(F) very similar estimates of sensitivity regardless of the specific nonparametric analysis used.

All three of the above nonparametric measures were used to calculate Mini-PROMS sensitivity to explore whether they (1) demonstrated construct validity, quantified as the correlation between years of musical training and each measure, and (2) whether they provided similar estimates of sensitivity. When calculating *d_a_* and *d′_p_*, proportions of 0 and 1 were first converted to 1/2 N and 1–1/2 N, respectively. All three nonparametric measures were significantly and similarly correlated with years of musical training (AUC: *r* = 0.549, *p* < 0.0001; *d_a_*: *r* = 0.520, *p* < 0.0001; *d′_p_*: *r* = 0.537, *p* < 0.0001), demonstrating adequate construct validity [Fig. 4(A)–4(C)], with effect sizes slightly stronger than the standard *d′* or composite score measures (cf. Fig. 1). Indeed, each of the three measures was highly correlated with the other two, with correlation coefficients (*r*) all exceeding 0.9 [Figs. 4(D)–4(F)]. Unlike the weighted composite score, which conflates sensitivity with confidence, all three nonparametric estimates were consistent with one another at quantifying musical competence. Supplementary Fig. 1 further demonstrates how participants with high nonparametric measures of sensitivity can have quite different weighted composite scores. The average Mini-PROMS performance score (with SD) for the AUC was 0.765 (0.12) and the values of *d_a_* and *d′_p_* were 1.19 (0.63) and 1.23 (0.65), respectively.

## 4. Discussion

The results show that the two most commonly used methods for scoring the PROMS, the composite score and weighted *d′*, are problematic because they are confounded by response bias. We found that unweighted *d′*, calculated by assuming equal variances, is also not a bias-free estimate of sensitivity. Our results indicate that a simple nonparametric *d′* score can provide a bias-free estimate of Mini-PROMS performance. Note that while the nonparametric SDT measures are preferable, the uneven number of trials across subtests means that melody and accent performance will influence overall performance slightly more than tuning and tempo.

When evaluating overall response bias, *c*, we found that response bias varied systematically with years of musical training for the total Mini-PROMS, the melody subtest, and the tuning subtest. Specifically, people with the least amount of musical training tended to respond “same” more often than “different.” This bias is somewhat expected, as people with fewer years of musical training may not be able to discern changes in the melodies as well as people with more musical training. Similar trends have been found for the Montreal Battery of Evaluation of Amusia (MBEA), when comparing people with a disorder in fine-grained pitch and melody perception, amusia, with controls matched in terms of years of musical training (Whiteford and Oxenham, 2017).

All three nonparametric measures of Mini-PROMS performance that we tested, AUC, *d_a_*, and *d′_p_*, demonstrated adequate construct validity, quantified as the correlation between the nonparametric performance and years of musical training. Although any of these three measures are suitable alternatives for measuring Mini-PROMS performance, researchers may find *d′_p_* to be the preferred method. First, *d′_p_* has already been used as a method for scoring the Micro-PROMS (Strauss *et al.*, 2023). Second, the calculation is simple and can be done with commonly used programs that are widely accessible, such as Microsoft Excel or Google Spreadsheets. We provide spreadsheet examples for this calculation on the Open Science Framework (Whiteford *et al.*, 2023). For any individuals with proportions of 0 or 1, we recommend using one of the established correction methods, such as replacing them with 1/2 N and 1–1/2 N, respectively (Macmillan and Creelman, 2005).

Consistent with previous methods of scoring the PROMS, there was wide variability in performance amongst people with similar amounts of self-reported musical training (Law and Zentner, 2012; Mankel and Bidelman, 2018), confirming the existence of “musical sleepers” (non-musicians with exceptional music perception skills) and “sleeping musicians” (people with extensive amounts of musical training who perform poorly on the PROMS), without the risk posed by the traditional composite method, which could mistakenly identify a musician with excellent perceptual skills as a “sleeping musician” even with every trial answered correctly, if they never use the extreme ends of the response scale.

Although the present study is limited to the Mini-PROMS version of the PROMS, the issue that the composite score is conflated by confidence applies to all versions. We therefore recommend the use of any of the nonparametric measures of sensitivity tested here for all PROMS tests, as they remain valid whether or not parametric assumptions hold for the underlying data.

## SUPPLEMENTARY MATERIAL

See the supplementary material for visualization of the relationship between the non-parametric measures and the Mini-PROMS composite score.

## Data Availability

The data that support the findings of this study are openly available in the Open Science Framework (Whiteford *et al.*, 2023).
